# Genome-Wide Association Study Reveals Novel Marker-Trait Associations (MTAs) Governing the Localization of Fe and Zn in the Rice Grain

**DOI:** 10.3389/fgene.2020.00213

**Published:** 2020-04-22

**Authors:** Haritha Bollinedi, Ashutosh Kumar Yadav, K. K. Vinod, S. Gopala Krishnan, Prolay Kumar Bhowmick, M. Nagarajan, C. N. Neeraja, Ranjith Kumar Ellur, Ashok Kumar Singh

**Affiliations:** ^1^Division of Genetics, ICAR-Indian Agricultural Research Institute, New Delhi, India; ^2^ICAR-Indian Agricultural Research Institute, Rice Breeding and Genetics Research Centre, Aduthurai, India; ^3^ICAR-Indian Institute of Rice Research, Hyderabad, India

**Keywords:** Fe, Zn, biofortification, GWAS, donors, rice, milled rice, brown rice

## Abstract

Micronutrient malnutrition due to Fe and Zn, affects around two billion people globally particularly in the developing countries. More than 90% of the Asian population is dependent on rice-based diets, which is low in these micronutrients. In the present study, a set of 192 Indian rice germplasm accessions, grown at two locations, were evaluated for Fe and Zn in brown rice (BR) and milled rice (MR). A significant variation was observed in the rice germplasm for these micronutrients. The grain Fe concentration was in the range of 6.2–23.1 ppm in BR and 0.8–12.3 ppm in MR, while grain Zn concentration was found to be in the range of 11.0–47.0 ppm and 8.2–40.8 ppm in the BR and MR, respectively. Grain Fe exhibited maximum loss upon milling with a mean retention of 24.9% in MR, while Zn showed a greater mean retention of 74.2% in MR. A genome-wide association study (GWAS) was carried out implementing the FarmCPU model to control the population structure and kinship, and resulted in the identification of 29 marker-trait associations (MTAs) with significant associations for traits viz. FeBR (6 MTAs), FeMR (7 MTAs), ZnBR (11 MTAs), and ZnMR (5 MTAs), which could explain the phenotypic variance from 2.1 to as high as 53.3%. The MTAs governing the correlated traits showed co-localization, signifying the possibility of their simultaneous improvement. The robust MTAs identified in the study could be valuable resource for enhancing Fe and Zn concentration in the rice grain and addressing the problem of Fe and Zn malnutrition among rice consumers.

## Introduction

In the human body, iron (Fe) and zinc (Zn) are the two most abundant trace minerals. An average adult human with a body weight of 65 kg has about 3–4 g of Fe and 1.5–2.5 g of Zn (King et al., 2006; Wood et al., 2006). Fe is essential for the synthesis of oxygen-transporting proteins *viz.* hemoglobin and myoglobin and is also an integral part of the enzymes involved in energy production and the maintenance of immune functions (Roeser, 1986; Stoltzfus, 2001). Zn is a pre-requisite for biological functions like gene expression, cell division, cell development, reproduction, and immunity (Brown et al., 2001, 2004). Fe and Zn are commonly viewed together in human nutrition, as they can be obtained through common dietary sources, and their absorption or inhibition is believed to be affected by similar factors (Lim et al., 2013). Fe deficiency in humans is related to increased risk of maternal mortality, anemia, premature births, low birth weight, and impaired cognitive and motor development (Bouis, 2003). Severe or clinical Zn deficiency is a condition associated with short stature, immune system dysfunction, hypogonadism, skin disorders, anorexia, delayed wound healing, skeletal abnormalities, and cognitive dysfunction (Prasad, 1991; Salgueiro et al., 2000).

Micronutrient malnutrition popularly known as “hidden hunger,” primarily attributed to Zn and Fe deficiency, is evolving as a global pandemic with serious health effects. The situation is more grim in developing countries and has become a fundamental limitation in achieving the Sustainable Development Goals (SDGs) in these countries. Mineral deficiency disorders caused by Fe and Zn deficiency are the primary reasons for the reduced work productivity in developing countries, which further affects the gross national product. Fe deficiency is recognized as the most widespread nutritional disorder with about 1.6 billion people suffering from iron deficiency anemia (IDA) globally. Additionally, Zn deficiency is ranked as the 5th major health risk factor in developing countries, affecting nearly two billion people worldwide (Zhang et al., 2018). The loss incurred due to Zn deficiency amounts to almost 16 million global disability-adjusted life years (DALYs) (Caulfield et al., 2004; Black et al., 2008).

The dietary habit of the majority of the population in developing countries as well as of a significant portion of the world populace is cereal based. In general, cereal foods are low in micronutrient content, especially when grains are consumed after extensive processing. Further, when cereal based diets are not balanced with other supplementary food items, there can be a severe deficiency of micronutrient nutrition leading to hidden hunger. Although the grain Fe and Zn concentrations in the brown rice (BR) of the modern-day high yielding rice cultivars are in the range of 6.3–24.4 mg/kg and 15.3–58.4 mg/kg, respectively (Gregorio et al., 2000), much of which are lost upon polishing, retaining a maximum of 2 mg/kg of Fe and 12 mg/kg of Zn in the grains that are consumed (Pradhan et al., 2020). Therefore, genetic fortification of rice grain becomes the most viable solution to address the challenge of micronutrient malnutrition. To begin with, identification of molecular players affecting the mineral uptake and transport, and understanding the genes and pathways governing mineral homeostasis and localization, would assist in devising targeted breeding strategies for biofortification.

A genome-wide association study (GWAS) provides an opportunity to utilize the tremendous genetic diversity of rice preserved among the landraces, to unravel the molecular mechanisms of complex traits, like mineral uptake and their grain accumulation. The method uses numerous historic recombination in a large natural population that offers the potential to localize the trait genetic determinants effectively to a narrower region. Further, application of single nucleotide polymorphism (SNP) in a GWAS study provides dense coverage of the entire genome, which would help identify the functional variation governing the trait. We used a diverse set of 192 rice germplasm accessions including landraces, popular varieties, Basmati accessions, and breeding lines collected from all over India in a GWAS to analyze the morphological variation for grain Fe and Zn content across two diverse locations, and to identify the marker-trait associations (MTAs)/genomic regions associated with Fe and Zn content in rice grain, and elite parents that can serve as donors in rice biofortification programs.

## Materials and Methods

### Plant Material and Field Experiment

The study comprised of an association mapping panel of 192 rice accessions collected from different parts of India and maintained at the Division of Genetics, ICAR-Indian Agricultural Research Institute (ICAR-IARI), New Delhi, India. The complete set of genotypes were evaluated at two locations *viz*. the ICAR-IARI farm (28.04°N; 77.12°E, 228 m) in New Delhi during the wet season (WS) of 2017, and the Rice Breeding and Genetics Research Centre (RBGRC) farm (11° 00′N; 79° 28′E, 19.5 m) in Aduthurai, Tamil Nadu during the dry season (DS) of 2017–2018. The soils from the IARI farm are of a sandy loam type with a pH of 7.4–7.8 with an average available Fe and Zn content of 12.3 and 3.3 mg/kg, respectively, while the soils from Aduthurai are typical haplusterts with a pH of 7.2 and mean available Fe and Zn content of 12.5 and 0.7 mg/kg, respectively. An augmented randomized complete block design was adopted for field evaluation. Similar agronomic practices were followed at both locations. The seeds were germinated on a raised seedbed to ensure uniform germination, and 28-day old seedlings were transplanted into a puddled rice field. Each genotype was planted in two rows of 3 m length with a 20 cm spacing between rows and 15 cm between plants. The recommended package of practices was adopted to ensure good crop stand. At maturity, plants were selected from the middle of the rows, and the grains were harvested, threshed, and dried to a moisture content of ≤13% and stored in zip lock bags at room temperature until analysis.

### Analysis of Grain Fe and Zn Concentration

Fe and Zn content of the samples was estimated in parts per million units (mg/kg) from both BR and milled rice (MR) using an energy dispersive X-ray fluorescence spectrometer (ED-XRF) (X-Supreme 8000, Oxford Instruments, CA, USA). Grain samples from each genotype was dehusked using a palm de-husker, and the BR was cleaned thoroughly and analyzed directly in ED-XRF. The samples were further milled in a non-ferrous, non-zinc rice polisher (Mini Lab Rice Polisher Model K-710, Krishi International, Hyderabad, India) to avoid contamination with the metals during polishing. The milled samples were further cleaned with a non-shredding tissue paper to remove all the residual bran, and whole grains were used for analysis in ED-XRF. Three independent samples drawn from the bulk sample were analyzed for Fe and Zn.

### DNA Isolation and SNP Genotyping

Genomic DNA was isolated from the 192 germplasm accessions using young leaves at 30 days after transplanting by adopting the CTAB method (Murray and Thompson, 1980). The quality of the DNA was assessed on a 0.8% agarose gel and was further quantified using a nano-drop spectrophotometer (NanoDrop^TM^ 2000/2000c, Thermo Fisher Scientific, DE, United States). High throughput genotyping was carried out using a custom-designed 50 K SNP chip. The chip was based on single-copy genes, covering all 12 rice chromosomes with an average interval of fewer than 1 kb between adjacent SNP markers (Singh et al., 2015). DNA amplification, fragmentation, chip hybridization, single-base extension through DNA ligation, and signal amplification were carried out as detailed in Singh et al. (2015).

### Population Structure and Linkage Disequilibrium Analyses

The number of subgroups in the association mapping panel was estimated using both a model-based approach using STRUCTURE 2.3.4 software (Pritchard et al., 2000) and principal component (PC) analysis. For the model-based analysis, a Bayesian model approach using an ancestry model of ADMIXTURE (Alexander et al., 2009) and a frequency model assuming correlated allele frequencies among the sub-populations was used. For this, a subset of 5,000 genome-wide SNP markers with minor allele frequency (MAF) ≥0.05 and missing data <20% was used. The analysis was run with an assumed number of subgroups (*K*) ranging from 1 to 10 and with each *K* replicated 10 times. For each run, 100,000 burn-in steps followed by 100,000 Markov Chain Monte Carlo simulations were implemented. The optimum number of *K* was determined according to Evanno et al. (2005) embedded in STRUCTURE HARVESTER (Earl and vonHoldt, 2012) by plotting the *ad hoc* statistic Δ*K* against the natural logarithms of probability data [LnP(*K*)]. PCA analysis, which was incorporated in the package “GAPIT” (genomic association and prediction integrated tool) running under *R* environment (Lipka et al., 2012) was used. A significant number of PCs that are adequate to explain the population structure were determined based on the scree plot generated by GAPIT. Tukey’s multiple comparison test was implemented to assess the significance of the difference in means of sub-populations. The extent of linkage disequilibrium between the SNP markers was analyzed by calculating the *r*^2^ values in TASSEL v5.2.20 (Bradbury et al., 2007). Only *r*^2^ values with *p* < 0.05 within each chromosome were considered for linkage disequilibrium (LD) decay analysis. Marker pairs were clustered into 5 kb bins, and the average *r*^2^ value of each bin was estimated and plotted against the distance. The physical distance at which the *r*^2^ value dropped to half of its average maximum value was considered an LD decay rate (Huang et al., 2010).

### GWAS Analysis

For GWAS analysis, the data obtained from 50,000 SNP markers was filtered for MAF ≥0.05 and maximum missing sites per SNP <20% and maximum missing sites per genotype <20%. A total of 31,132 SNPs remained after filtering, and were used for further analysis. One accession was dropped from the analysis due to the poor quality of the genotypic data. The data was analyzed using multiple statistical models *viz*. general linear model (GLM), mixed linear model (MLM), and fixed and random model circulating probability unification (FarmCPU; Liu, 2015; Liu et al., 2016). The efficiency of these models in controlling the familial relatedness and population structure was assessed by comparing the quantile–quantile (Q–Q) plots obtained through plotting observed *p*-values against the expected values. In each case, significant MTAs were identified after a modified Bonferroni multiple test correction calculated from the reciprocal of total number of markers used for analysis [*p* < 3.21E-05; -log_10_(*p*) > 5.79]. The percentage of phenotypic variance explained (PVE) by individual SNP was calculated through the single-marker analysis.

### Assessment of the Novelty of Identified MTAs

The novelty of MTAs identified in the study was determined through the comparison of the physical positions, with those of previously reported quantitative trait loci (QTLs) for Fe and Zn. In addition to the literature survey, the gramene QTL database^1^ and QTL annotation rice online database (QTARO)^2^ were searched to identify the physical locations of the previously reported QTLs. The candidate genes in the vicinity of the MTA region were determined using the genome browser of the Rice Annotation Project Database (RAP-DB)^3^.

## Results

### Population Structure

**FIGURE 1:**
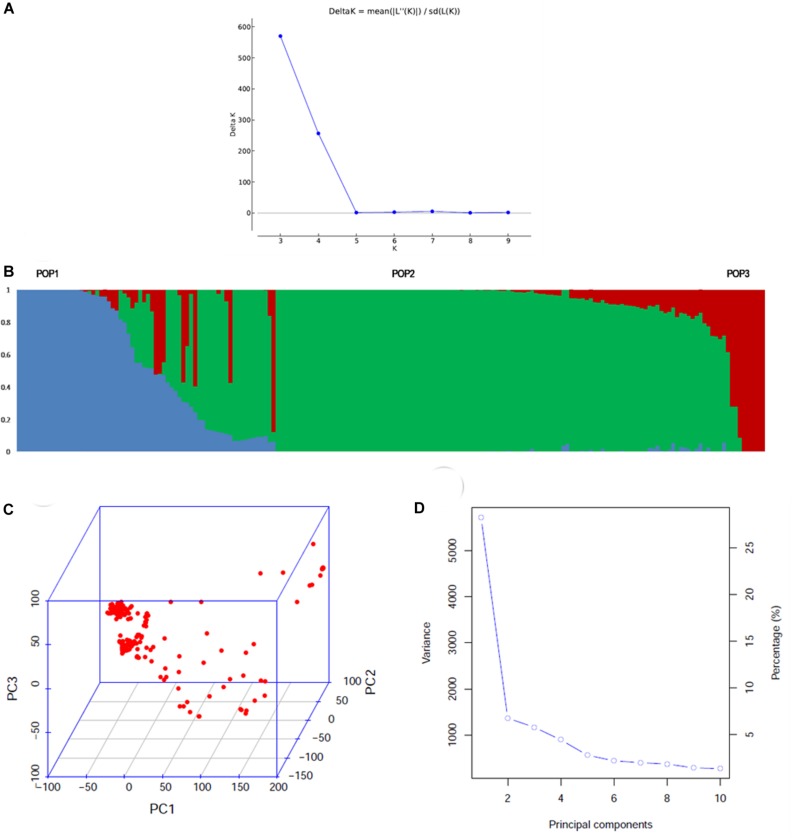
Population structure of the association mapping panel comprising of 192 genotypes analyzed by model based and PCA based approaches. **(A)** Δ*K* plot depicting three subgroups in the population by Evanno’s method. The highest Δ*K* was 580 at *K* = 3, **(B)** the bar plot showing the three sub-populations identified. POP2 was the largest and showed remarkable admixture with POP1. POP3 was the smallest group, which showed less admixing with POP1 **(C)** 3D graph depicting the distribution of accessions along the three PCs **(D)** scree plot depicting the number of significant PCs. There were three PCs that explained a cumulative variation of ∼40%.

**FIGURE 2:**
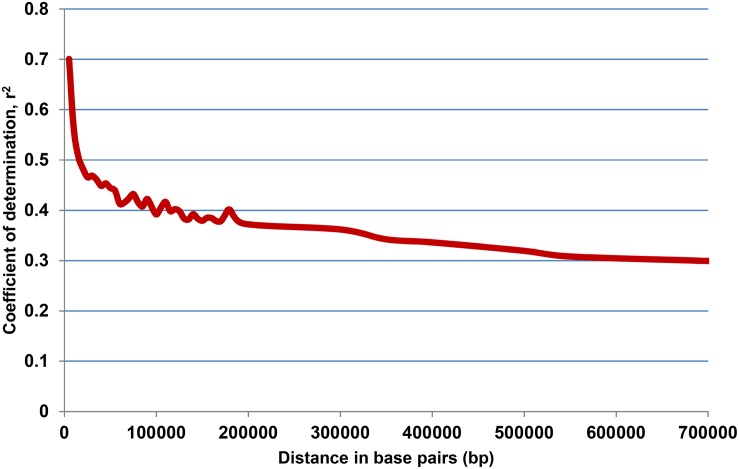
Linkage disequilibrium (LD) decay plot of the association mapping panel derived from 31,132 SNPs, plotted against physical distance in base pairs (bp) and co-efficient of determination (*r*^2^).

Model-based simulation of population structure showed a sharp peak at *K* = 3 when the number of clusters was plotted against Δ*K*, depicting the presence of three sub-populations in the panel (Figure 1A). The sub-populations are denoted as POP1, POP2, and POP3. Figure 1B shows a representative picture of the population structure in which each individual is indicated by a vertical bar, which is divided into segments based on its estimated membership fractions in sub-populations. POP2 was the largest and constituted 73.5% of the panel numbering 139 accessions, of which 74 were pure types (includes accessions with <5% of admixture) and 65 were admixture types. POP1 consisted of 35 accessions including 13 pure types and 22 admixture types. Unique landraces from the Jammu and Kashmir region like Begum, BalaKoan, Buta Baber, Mehvan, etc., were all included in POP1. In addition, it also consisted of aromatic rice accessions including the traditional Basmati varieties like Basmati 370, Super Basmati, Type 3 and short grain aromatic accessions like Kalanamak, Chittimutyalu, etc. All the evolved varieties like MTU1001, ADT 39, IR 70, MAS946-1, Improved Sabarmati, and breeding lines like PRR 109, PRR 127, etc., were grouped into POP2. POP3 is the smallest, with 15 accessions, of which six were pure types and the rest were admixture types. Two accessions, PDKV-Chinoor 2 and SAF 1221-83, were admixtures and were therefore not included in any of the sub-groups. The fixation index (*F*_st_) was 0.74, 0.74, and 0.68 for the sub-populations POP1, POP2, and POP3, respectively, and the expected heterozygosity or the average distance between individuals within a sub-population was 0.15, 0.18, and 0.17. The allelic frequency divergence of POP1 from POP2 was 0.42, and from POP3 was 0.34, while between POP2 and POP3 it was 0.39. The PCA based lookout for the presence of structure in the population also indicated significance of three PCs in the population, explaining about 39.6% of total variation (Figure 1C). The scree plot (Figure 1D) showed that the first PC explained the highest variation of 26.7% followed by second and third PCs explaining 6.8 and 6.0% of the total variation, respectively. Admixture analysis revealed that about 53.60% of the accessions (103/192) showed 0–5% of admixture, 12% of the accessions (23/191) showed 5–20% of the admixture, while the remaining 34% of the accessions depicted >20% of the admixture. LD analysis in the association panel revealed a highest average *r*^2^ of 0.7 at shorter distance of 5 kb, and it decreased to half its value at around 350 kb (Figure 2).

### Variation for Grain Mineral Micronutrients in the Association Panel

The grain Fe and Zn contents exhibited significant variation in the association panel (Table 1). In New Delhi, during WS 2017, Fe concentration in BR (FeBR) varied from 6.5 (Jayati) to 23.1 (Shah Pasand) mg/kg with a mean of 12.7 ± 0.2 mg/kg, while in milled rice (FeMR) it ranged from 0.8 (Arupathaam Kuruvai) to 12.3 (IC-2127) mg/kg with an average of 3.6 ± 0.1 mg/kg. In Aduthurai, the variation for FeBR was similar to that of New Delhi and ranged from 6.2 (Khara Munga) to 21.4 (PRR 109) mg/kg with a mean value of 10.6 ± 0.2 mg/kg. The FeMR exhibited a comparatively narrow range of 0.9 (Ramachandi) to 5.6 (PRR 109) mg/kg with a mean of 2.6 ± 0.1 mg/kg. Alternatively, grain Zn content in BR (ZnBR) varied from 13.0 (Sagar samba) to 46.2 (Karuppunel) mg/kg in the New Delhi location, while in Aduthurai its range was 11.0 (Samanta and OYR 128) to 47 (Karuppunel) mg/kg. In MR, however, the mean value for grain Zn (ZnMR) was 18.0 ± 0.4 mg/kg in New Delhi with a range between 8.2 (Sagar Samba) and 40.9 (Karuppunel) mg/kg, while in Aduthurai, it varied between 8.5 (Bhadrakali) and 40.8 (Karuppunel). The average grain ZnMR in Aduthurai was 16.5 ± 0.4 mg/kg, which was much closer to that observed in the New Delhi conditions.

**TABLE 1:** Basic statistics of the grain Fe and Zn content in the association mapping panel used in the study, at two diverse locations, New Delhi and Aduthurai.

Trait	New Delhi			Aduthurai		
		
	Mean ± SE	Range	Variance	Mean ± SE	Range	Variance
FeBR	12.70.2	6.5–23.1	10.6	10.60.2	6.2–21.4	7.9
FeMR	3.60.1	0.8–12.3	3.3	2.60.1	0.9–5.6	1.0
ZnBR	22.90.4	13.0–46.2	32.9	21.70.4	11–47	32.5
ZnMR	18.00.4	8.2–40.9	28.9	16.50.4	8.5–40.8	25.9

*FeBR, Fe content in brown rice; FeMR, Fe content in milled rice; ZnBR, Zn content in brown rice; ZnMR, Zn content in milled rice; SE, standard error.*

The association panel depicted significant variation for percent retention of mineral micronutrients upon polishing. A substantial quantity of Fe was lost upon polishing and its retention on milling ranged from as low as 5.4% (Aziz Beoul) to 89.1% (OYR 128) with a mean retention of 24.9% in the MR. When compared to grain Fe, less polishing loss of grain Zn was evident with a retention ranging from 45.8% (OYC 183) to as high as 97.3% (Kalanamak). Of the 192 accessions analyzed, as many as 75 genotypes exhibited a retention of ≥80% of grain Zn.

**FIGURE 3:**
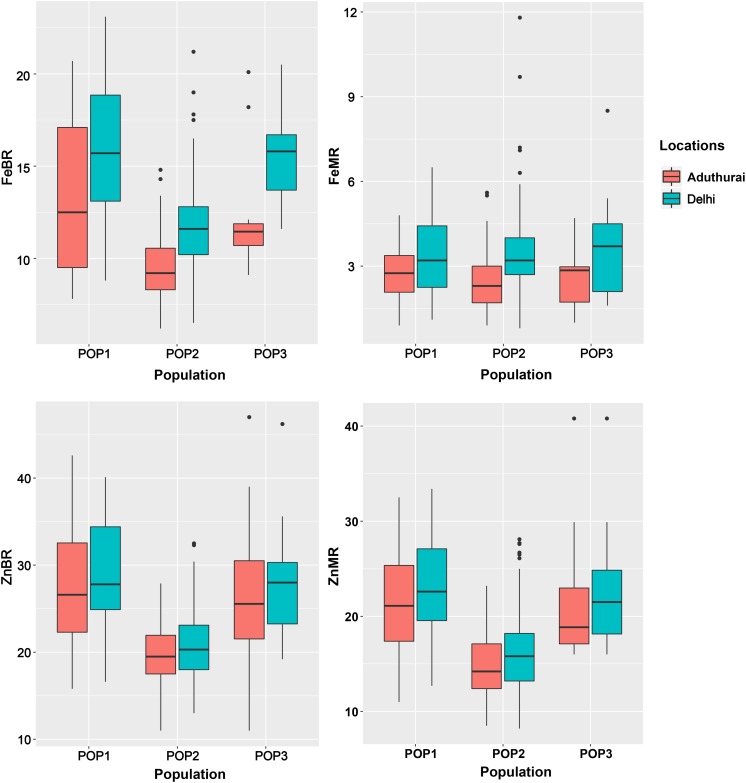
Boxplots showing distribution of grain Fe and Zn content among the sub-populations POP1, POP2, and POP3. FeBR, Fe concentration in brown rice; FeMR, Fe concentration in milled rice; ZnBR, Zn concentration in brown rice; ZnMR, Zn concentration in milled rice.

The sub-populations do not differ significantly for grain Fe and Zn concentration except for the POP2 having slightly lower mean values than POP1 and POP3 (Table 2). POP3 showed a significant difference for the traits between the locations with highest values recorded at the New Delhi location (Figure 3). Population structure accounted for a variation in Fe and Zn concentration ranging from 2.7 (FeMR) to 34.2% (ZnBR) at the New Delhi location and 2.1 (FeBR) to 32.4% (ZnMR) at the Aduthurai location.

**TABLE 2:** Mean and range values for Fe and Zn content among the three sub-populations of the association mapping panel for the two locations, New Delhi and Aduthurai.

Traits	POP1		POP2		POP3		*R*^2^
			
	Mean ± SD	Range	Mean ± SD	Range	Mean ± SD	Range	
**New Delhi**							
FeBR	15.93.9	8.8–23.1	11.62.3	6.5–21.2	15.42.5	11.6–20.5	31.6
FeMR	3.41.4	1.1–6.5	3.51.5	0.8–11.8	3.81.9	1.6–8.5	2.7
ZnBR	28.36.5	16.6–40.1	20.93.8	13–32.5	28.76.6	19.2–46.2	34.2
ZnMR	22.85.1	12.7–33.4	16.24.1	8.2–28.1	22.76.5	16–40.8	30.0
**Aduthurai**							
FeBR	13.54.2	7.8–20.7	9.72.0	6.2–21.4	12.13.2	9.1–20.1	25.9
FeMR	2.80.9	0.9–4.8	2.41.0	0.9–5.6	2.71.2	1–4.7	2.1
ZnBR	27.66.6	15.8–42.6	20.03.8	11–31.4	26.79.0	11.0–47.0	30.2
ZnMR	21.45.5	11.0–32.5	14.83.5	8.5–26.4	21.56.7	16–40.8	32.4

*R^2^ indicates the percentage of total phenotypic variation explained by sub-populations.*

### Associations Among the Mineral Micronutrients

**FIGURE 4:**
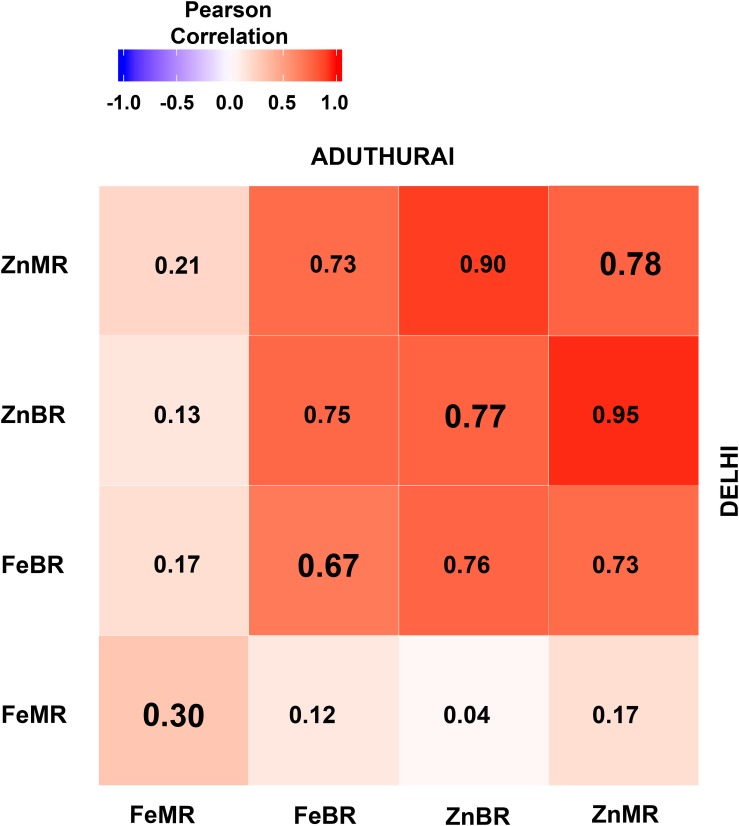
Correlations of Fe and Zn between the locations (diagonal elements) and independent locations at New Delhi (upper diagonal) and Aduthurai, Tamil Nadu (lower diagonal). FeBR, Fe concentration in brown rice; FeMR, Fe concentration in milled rice; ZnBR, Zn concentration in brown rice; ZnMR, Zn concentration in milled rice.

Grain mineral micronutrient content between two locations were significantly correlated (*p* > 0.001). Strong positive correlations were observed for ZnMR (*r* = 0.78), ZnBR (*r* = 0.77), and FeBR (*r* = 0.67) while FeMR (*r* = 0.30) indicated a moderate association. Trait wise within location correlation analysis indicated that grain Zn in BR was significantly correlated with Zn concentration in MR with high positive values in both New Delhi (*r* = 0.95; *p* > 0.0001) and Aduthurai (*r* = 0.90; *p* > 0.0001). However, FeBR showed non-significant association with FeMR. Nevertheless, FeBR exhibited significant positive correlation with ZnBR as well as with ZnMR (Figure 4).

### Detection of Stable and Environment-Specific MTAs by GWAS

**FIGURE 5:**
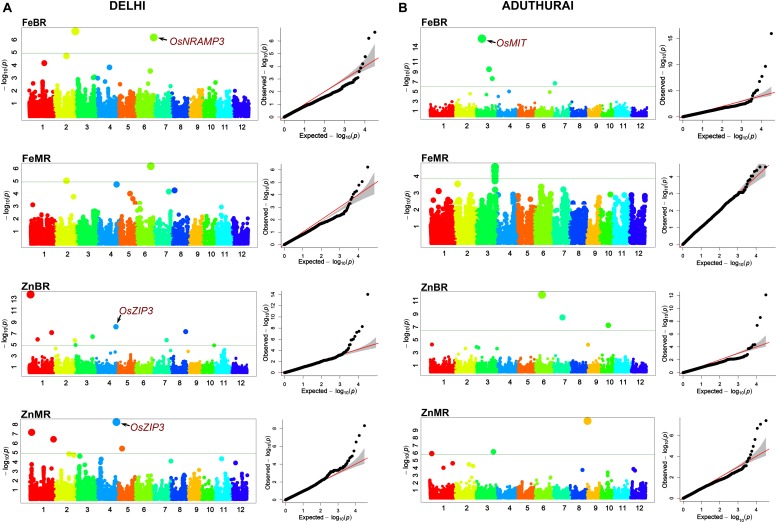
Manhattan plots and Q–Q plots derived through FarmCPU model depicting the significant marker trait associations on 12 chromosomes of rice for the traits analyzed at New Delhi **(A)** and Aduthurai **(B)** locations.

Association analysis was performed separately for the two locations. The Q–Q plots generated through the FarmCPU model depicted less deviation of the observed *p*-values from the expected *p*-values and was therefore chosen as the best fit (Figure 5). A total of 29 QTLs located along 9 out of 12 chromosomes were identified for the four traits analyzed in two environments (Table 3). Eleven were major effect QTLs with a PVE more than 20%, 10 were moderate effect QTLs (PVE 20–10%), while the remaining seven were minor effect QTLs (PVE < 10%). One MTA, *qZnMR1.1* showed moderate effect at the New Delhi location and minor effect at the Aduthurai location. Chromosome 3 had the highest number of 11 MTAs, followed by chromosome 1 (six MTAs), chromosomes 2, 6, and 7 (two MTAs each), and chromosomes 8, 9, and 10 (one MTA each). No MTAs affecting Fe and/or Zn concentration in rice grains were found on chromosome 5, 11, and 12. Manhattan plots depicting the significant SNP markers above the modified Bonferroni threshold are provided in Figure 5.

**TABLE 3:** Details of the marker-trait associations (MTAs) mapped for grain Fe and Zn content at two different locations, New Delhi and Aduthurai.

SNP	Allele		Chromosome	Position	Probability	Trait	Site	QTL	*R*^2^	*a*_*i*_	Previous report
	
	Favorable allele	Alternate allele									
AX-95918814	A	G	1	3,565,733	6.19E-08	ZnMR	DEL	qZnMR1.1	9.3	1.7	*
AX-95918814	A	G	1	3,565,733	6.19E-08	ZnMR	ADT	*qZnMR1.1*	10.4	1.8	*
AX-95934119	T	C	1	3,568,378	8.72E-15	ZnBR	DEL	*qZnBR1.1*	12.4	3.4	*
AX-95919006	A	C	1	15,673,604	8.88E-07	ZnBR	DEL	*qZnBR1.2a*	45.5	4.8	Stangoulis et al., 2007
AX-95917932	G	A	1	39,382,522	5.63E-08	ZnBR	DEL	*qZnBR1.2b*	40.1	4.3	*
AX-95918225	G	T	1	41,121,295	3.33E-07	ZnMR	DEL	*qZnMR1.2*	17.7	4.2	*IRO2*
AX-95921433	C	A	2	34,958,759	2.01E-07	FeBR	DEL	*qFeBR2.1*	6.8	1.5	Swamy et al., 2018
AX-95965057	A	G	2	35,093,342	1.35E-06	ZnBR	DEL	*qZnBR2.1*	26.4	5.0	Swamy et al., 2018
AX-95922070	A	G	3	10,164,543	1.06E-16	FeBR	ADT	*qFeBR3.1*	53.3	4.1	*MIT*, *NAS1*, *NAS2*
AX-95962496	A	C	3	22,193,098	2.03E-10	FeBR	ADT	*qFeBR3.2*	2.1	0.2	*
AX-95921838	G	T	3	27,810,157	1.71E-08	FeBR	ADT	*qFeBR3.3*	2.1	0.1	*
AX-95923364	C	T	3	29,493,448	2.95E-07	ZnBR	DEL	*qZnBR3.1*	39.9	4.0	Anuradha et al., 2012a
AX-95921738	T	G	3	30,176,449	5.86E-07	ZnMR	ADT	*qZnMR3.1*	18.3	3.3	*
AX-95935621	G	A	3	32,326,592	4.79E-05	FeMR	ADT	*qFeMR3.1a*	12.8	0.4	*
AX-95950999	T	G	3	32,335,075	4.13E-05	FeMR	ADT	*qFeMR3.1b*	12.9	0.4	*
AX-95935460	G	A	3	32,374,286	8.90E-05	FeMR	ADT	*qFeMR3.1c*	12.3	0.4	*
AX-95924055	C	G	3	32,380,341	8.90E-05	FeMR	ADT	*qFeMR3.1d*	12.3	0.4	*
AX-95923317	T	C	3	32,380,432	2.61E-05	FeMR	ADT	*qFeMR3.1e*	13.7	0.4	*
AX-95923159	G	A	3	32,380,964	2.61E-05	FeMR	ADT	*qFeMR3.1f*	13.7	0.4	*
AX-95951158	A	G	4	32,811,874	5.24E-09	ZnBR	DEL	*qZnBR4.1*	47.6	6.3	*ZIP3*
AX-95951158	A	G	4	32,811,874	4.86E-09	ZnMR	DEL	*qZnMR4.1*	42.7	5.6	*ZIP3*
AX-95927387	A	G	6	11,840,203	8.42E-13	ZnBR	ADT	*qZnBR6.1*	44.9	7.4	*
AX-95928882	C	A	6	24,918,156	6.05E-07	FeMR	DEL	*qFeMR6.1*	3.2	0.6	*
AX-95956929	A	G	6	30,278,880	6.11E-07	FeBR	DEL	*qFeBR6.1*	45.2	2.7	*NRAMP3*
AX-95915606	A	T	7	2,636,599	1.66E-07	FeBR	ADT	*qFeBR7.1*	41.5	4.7	*
AX-95929962	T	G	7	15,616,980	2.71E-09	ZnBR	ADT	*qZnBR7.1*	16.5	3.4	Anuradha et al., 2012a
AX-95929638	T	A	7	22,727,786	1.23E-06	ZnBR	DEL	*qZnBR7.2*	33.4	6.6	Anuradha et al., 2012a
AX-95930744	C	A	8	25,586,491	3.74E-08	ZnBR	DEL	*qZnBR8.1*	2.3	0.6	Garcia-Oliveira et al., 2009
AX-95959928	T	C	9	849,821	4.76E-11	ZnMR	ADT	*qZnMR9.1*	7.9	2.2	*
AX-95932094	A	G	10	12,685,215	4.51E-08	ZnBR	ADT	*qZnBR10.1*	6.8	1.7	*

*SNP, single nucleotide polymorphic marker; DEL, New Delhi; ADT, Aduthurai; PVE, percentage variation explained; *a*_*i*_, additive effect of the favorable allele; FeBR, Fe content in brown rice; FeMR, Fe content in milled rice; ZnBR, Zn content in brown rice; ZnMR, Zn content in milled rice; asterisk indicates MTAs not reported so far.*

*FeBR:* A total of six MTAs affecting FeBR were identified in the study. Two MTAs were identified at the New Delhi location one each on chromosomes 2 and 6. The MTA on chromosome 6 had a major effect with a PVE of 45.2%, and its favorable allele A at the SNP marker locus AX-95956929 had an additive effect of 2.7 mg/kg on the trait. The MTA on chromosome 2 was a minor effect one with a PVE of only 6.8%. Four MTAs for FeBR were identified at the Aduthurai location, two of them were major effect associations with a PVE as high as 53.3% (*qFeBR3.1*) and 41.5% (*qFeBR7.1*) and an additive effect of 4.1 and 4.7, respectively.

*FeMR:* For FeMR, no MTAs were detected at Bonferroni threshold. However, by adopting a false discovery rate (FDR) threshold, seven MTAs were identified across two locations, of which six were moderate effect MTAs with a PVE ranging from 12.3 to 13.6%. All of these were detected at the Aduthurai location and were found clustered around the 32 Mb physical location on chromosome 3. Only one minor effect MTA was identified at the New Delhi location which explained 5.3% variation.

*ZnBR*: For this trait, a total of 11 MTAs were identified across two locations, eight under New Delhi conditions and three under Aduthurai conditions. The MTAs detected in New Delhi explained a PVE ranging between 12.4 (*qZnBR1.1*) and 47.6% (*qZnBR4.1*), of which six were major MTAs. The additive effect described by the favorable alleles of the MTAs was in the range of 3.4 to 6.6 mg/kg. At Aduthurai, three MTAs were recorded for ZnBR, one each on chromosomes 6, 7, and 10. The MTA on chromosome 6 was a major effect MTA and explained a PVE of 44.9%, which also recorded the highest additive effect of 7.4 mg/kg. Of the remaining two MTAs, the MTA on chromosome 7 was a moderate effect MTA with a PVE of 16.5%, while the one on chromosome 10 was a minor MTA with a PVE of 6.8%.

*ZnMR*: For the Zn concentration in MR, a total of five MTAs were identified, two each in the New Delhi and Aduthurai locations. One MTA, *qZnMR1.1* was commonly detected in both the sites with a PVE of 9.3 and 10.3% in New Delhi and Aduthurai, respectively. The PVE explained by the MTAs identified under the New Delhi condition ranged between 42.7 (*qZnMR4.1*) and 9.3% (*qZnMR1.1*), while those at the Aduthurai location explained a PVE between 18.3 (*qZnMR3.1*) and 7.9% (*qZnMR9.1*).

### Co-localization of MTAs

The MTAs, *qZnBR1.2a* and *qZnBR1.2b* identified for ZnBR at the New Delhi location were located in the same LD block and were co-localized on chromosome 1 at the Aduthurai location, six MTAs for FeMR namely *qFeMR3.1a*, *qFeMR3.1b*, *qFeMR3.1c*, *qFeMR3.1d*, *qFeMR3.1e*, and *qFeMR3.1f* were found to be in tight linkage on chromosome 3. Further, for the highly correlated traits such as ZnBR and ZnMR, co-localized MTAs were detected on chromosome 4 (*qZnBR4.1* and *qZnMR4.1*) as well as on chromosome 1 (*qZnBR1.1* and *qZnMR1.1*) under the New Delhi environment. Besides, *qZnBR4.1* and *qZnMR4.1* were found to share a common peak SNP. Between the environments, *qZnBR3.1* identified under New Delhi conditions for the trait ZnBR was found co-localized with eight other MTAs affecting the traits ZnMR (*qZnMR3.1*), FeBR (*qFeBR3.1*), and FeMR (*qFeMR3.1a*, *qFeMR3.1b*, *qFeMR3.1c*, *qFeMR3.1d*, *qFeMR3.1e*, and *qFeMR3.1f*) identified under Aduthurai conditions. Further, the *qFeBR6.1* identified at the New Delhi location was found co-localized with the *qZnBR6.1* identified at the Aduthurai location for the trait ZnBR. Additionally, the MTAs governing ZnBR, *qZnBR7.1* from the Aduthurai location and *qZnBR7.2* from the New Delhi location have also appeared co-localized on chromosome 7.

It was interesting to note that 12 of the 29 MTAs identified in this study were found to be in the vicinity of previously reported QTL regions and candidate genes. The MTA, *qFeBR3.1* was in close proximity to the known candidate genes *OsMIT*, *OsNAS1*, and *OsNAS2* on chromosome 3 and were located on the same LD block. Further, *qZnBR4.1* and *qZnMR4.1*, the co-localized MTAs associated with grain Zn concentration, were found linked to the reported candidate gene *OsZIP3*, while *qFeBR6.1* was found to be near the gene *OsNRAMP3* on chromosome 6.

## Discussion

Mineral micronutrient malnutrition is a widespread malady among the rice-eating populations, particularly in developing nations who cannot afford dietary diversity. Biofortification of popular rice varieties with mineral micronutrients, especially Fe and Zn, is a sustainable solution to tackle hidden hunger. However, polygenic inheritance of grain micronutrient accumulation in rice (Huang et al., 2015; Descalsota et al., 2018; Swamy et al., 2018) makes it relatively difficult to map multiple genes using a biparental population, when the genes have low individual effects and are sparsely distributed in the gene pool. A GWAS therefore offers a dual advantage of analyzing the extensive trait variation among the germplasm lines and identifying several genomic regions affecting the trait. In the present study, we analyzed a set of 192 rice germplasm accessions indigenous to different parts of India, for two important mineral micronutrients *viz*. Fe and Zn, in both BR and MR grown at two different locations, and found that the accessions possessed more extensive genetic diversity for both traits. Wide variability in rice accessions especially involving several landraces, has previously been reported particularly in BR (Anuradha et al., 2012b; Maganti et al., 2019). In this study, additionally, we demonstrated the existence of variation in MR which is more commonly consumed. In this case also, as reported earlier, we observed a pattern of polygenic inheritance of the Fe and Zn concentration from their normal distribution under both locations. This is in line with previous studies that demonstrated complex and multi-factorial inheritance of Fe and Zn in rice grain (Huang et al., 2015; Descalsota et al., 2018; Swamy et al., 2018).

A significant positive correlation between Fe and Zn in BR had been reported earlier (Stangoulis et al., 2007; Anuradha et al., 2012a) and together this study affirms the possibility of the simultaneous improvement of Fe and Zn in rice grain. As mentioned earlier, in rice grains, Fe and Zn are accumulated in the bran to the tune of about 30 and 6 mg/g, respectively (Bhosale and Vijayalakshmi, 2015), which accounts for the significant proportion of the mineral content in the grain. Additionally, the study also depicted strong association between Zn concentrations in BR and MR. It ascertains that the Zn concentration in BR is a fair indicator of its level in MR and can therefore be used as a selection criterion for the quick and non-destructive evaluation of Zn in the breeding populations targeted to improve Zn content in MR. In contrast, Fe concentration in BR was not found to be associated with Fe concentration in MR, signifying the practical need for enriching the Fe concentration of endosperm independent of its BR Fe concentration. When quantified, the percent retention upon polishing was found to be significantly low for Fe, in line with the previous reports that established localization of Fe in the embryo and aleurone layer (Prom-u-Thai et al., 2003; Choi et al., 2007; Kyriacou et al., 2014). Conversely, retention of Zn was significantly higher in the endosperm with moderate to minimal losses on polishing. However, examining carefully on an individual basis, the retention level varied across the genotypes, opening up the possibility of identifying genotypes with better nutrient retention in milled grains. Further studies are needed to investigate if this variation in percent retention upon polishing is due to differences in the ability of genotypes to translocate the mineral elements from aleurone to endosperm, or due to differences in the thickness of the aleurone layer.

### GWAS Identified Significant MTAs for Biofortification

Assessment of genetic diversity and population structure is an important pre-requisite in a GWAS and the presence of three sub-populations in our panel has been depicted by both PCA and STRUCTURE analyses. Variations observed among the three sub-populations for the Fe and Zn in both BR and MR at two locations, implied that grain micronutrients can only be subtly influenced by genetic grouping. The genotypes in the POP3 showed specific adaptation to location, with higher mean values recorded at the New Delhi location compared to the Aduthurai location. Huang et al. (2015) also reported the importance of population structure in determining the variation for the mineral micronutrients Fe and Zn and heavy metals like Pb, Cd, and Se in whole grain rice.

It has been proven that the GWAS accelerated the speed and accuracy of detecting QTLs and candidate genes in comparison to biparental linkage mapping. Several statistical models like GLM and MLM have been developed to control false positives and false negatives that arise due to familial relatedness and population structure. Ever since the publication of MLM, it has been popularly adopted for GWAS in crops, particularly, in rice (Zhang et al., 2014; Ya-fang et al., 2015; Wang et al., 2016). Nevertheless, MLM being a single locus method that allows testing of one marker locus at a time, had an inherent limitation in matching the real genetic architecture of the complex traits that are under the influence of multiple loci acting simultaneously (Kaler and Purcell, 2019). Multi-locus models like FASTmrEMMAa (Zhang et al., 2018), LASSO (Xu et al., 2017), BLASSO (Tamba et al., 2017), FarmCPU, pLARmEB (Zhang et al., 2018), and pKWmEB (Ren et al., 2018) are being used to overcome the limitation above. A few recent studies on plant height and flowering time (Wallace et al., 2016), ear traits (Zhu et al., 2018), and starch pasting properties (Xu et al., 2018) in maize, yield-related features in wheat (Ward et al., 2019), stem rot resistance in soybean (Wei et al., 2017), agronomic traits in foxtail millet (Jaiswal et al., 2019), and panicle architecture in sorghum (Zhou et al., 2019), have demonstrated the power of the FarmCPU model that uses both fixed effect and random effect models iteratively to effectively control the false discovery. In the present study, a comparison of Q–Q plots obtained through different models revealed FarmCPU as a best-fit model with improved power of test statistics. Our research forms the first report in rice that demonstrates the power of the FarmCPU over MLM.

Population size is another critical factor that determines the power of detection of QTLs in a GWAS (Garcia et al., 2005). In the present study, we identified significant MTAs even after adopting the highly stringent Bonferroni threshold, implying that the population size of 192 individuals is just enough for a GWAS in rice. Hoang et al. (2019) used a set of 180 individuals in a GWAS to identify QTLs affecting tolerance to water deficit while Descalsota et al. (2018) used a set of 144 MAGIC lines as a GWAS panel and mapped significant QTLs for agronomic and biofortification traits in rice. As rice is an autogamous species, it carries haplotype blocks larger than allogamous crops like corn. Yonemaru et al. (2012) demonstrated that size of the haplotype blocks in rice could vary widely, with a mean of ∼50 kb, while in corn it was ∼1 kb (Maldonado et al., 2019). Because of this, in crops like rice, a relatively smaller number of genotypes are required to cover a more significant proportion of the evolutionarily conserved genomic regions. Throughout the current decade, the use of GWAS has been particularly widespread in rice, helping to map QTLs related to traits such as agronomic characters (Zhang et al., 2014), plant height and grain yield (Ma et al., 2016), grain traits (Edzesi et al., 2016; Feng et al., 2016; Wang et al., 2016), panicle traits (Zhang et al., 2014), and milling quality (Qiu et al., 2015) to mention a few. Nevertheless, only a couple of studies reported a GWAS for mineral elements either in BR (Norton et al., 2014; Yang et al., 2018) or in MR (Descalsota et al., 2018).

In the present study, we have identified a total of 29 QTLs affecting Fe and Zn concentration in both BR and MR, holding significance in rice biofortification programs. Interestingly, 18 of the 29 MTAs were different from previous reports and can therefore be considered novel MTAs. The correlated traits like FeBR, ZnBR, and ZnMR shared common MTAs while uncorrelated features such as FeBR and FeMR did not share any co-localized MTAs. These co-localized MTAs can be targeted for the simultaneous improvement of Fe and Zn in rice grain. Further, the MTA, *qFeBR3.1* reported in this study on chromosome 3 was found in close proximity (222 kb) to the candidate gene, the mitochondrial iron transporter (*OsMIT*). Using knockdown mutants, *MIT* gene expression was shown to affect Fe localization in rice seeds (Bashir et al., 2013). Additionally, *qFeBR3.1* also shares the same LD block with the nicotianamine synthase (NAS) family protein *OsNAS1* (760 Kb) and *OsNAS2* (763 Kb). NAS family members catalyze the biosynthesis nicotinamine (NA) that acts a chelator of metal cations like Fe^2+^ and Fe^3+^ and play an essential role in both the short-distance as well as long-distance transport of metal cations (von Wiren et al., 1999; Takahashi et al., 2003). Overexpression of NAS genes resulted in several folds increase in Fe, Zn, and NA in rice grain (Lee et al., 2009; Johnson et al., 2011). The MTAs, *qZnBR4.1*, and *qZnMR4.1* on chromosome 4 were found to be co-localized with the *OsZIP3* gene, one of the members of the Zn-regulated transporter family proteins in rice. Initially, it was reported that the cation transporter proteins ZIP1 and ZIP3 might be involved in the uptake of Zn from soil (Ramesh et al., 2003; Bashir et al., 2012). Subsequently, Sasaki et al. (2015) observed that *OsZIP3* is localized in the nodes, and they further demonstrated that the OsZIP3 protein is responsible for unloading the Zn from the xylem of enlarged vascular bundles in nodes. In addition to these vital candidate genes, 7 of the 29 MTAs were within the intervals of previously mapped QTLs (Table 3).

### Potential Donor Germplasm Identified

We have identified four accessions (Table 4) with grain Zn concentration in MR > 28 mg/kg, a target set by the HarvestPlus program for rice bio-fortification (Bouis and Saltzman, 2017). These accessions showed consistent high grain Zn in both the environments tested. Additionally, several specifically adapted germplasm were also identified in the study. However, for the Fe concentration in MR, only one accession (IC-2127) showed >12 mg/kg Fe, as per the HarvestPlus target, specifically in the New Delhi location. Our data is in agreement with the previous studies that reported an average of only 2 mg/kg Fe in MR (Johnson et al., 2011; Yuan et al., 2013), indicating the limited scope for the improvement of Fe concentration in rice using conventional breeding approaches. Genetic engineering, especially the recent techniques of genome editing like CRISPR/Cas9 and transcription activator-like effector nucleases, offers viable alternatives to traditional methods for Fe bio-fortification in rice grain (Goto et al., 1999; Wirth et al., 2009; Masuda et al., 2012; Trijatmiko et al., 2016).

**TABLE 4:** The average grain content (both brown and milled rice) of Fe and Zn (mg/kg) in elite accessions identified from the association mapping panel to be used as donors in bio-fortification programs.

Lines	New Delhi				Aduthurai			
		
	FeBR	FeMR	ZnBR	ZnMR	FeBR	FeMR	ZnBR	ZnMR
Karuppunel	16.2	5.4	46.2	40.9	11.9	2.6	47.0	40.8
Budgi	17.8	1.4	35.6	31.6	17.4	2.0	35.4	30.2
Mehvan green	20.5	1.6	35.6	28.5	20.1	2.4	39.0	29.9
Mehvan purple	21.7	4.2	37.0	33.4	17.1	3.3	33.8	29.6

*FeBR, Fe content in brown rice; FeMR, Fe content in milled rice; ZnBR, Zn content in brown rice; ZnMR, Zn content in milled rice.*

## Data Availability Statement

The SNP data has been deposited into a publicly accessible repository held under ICAR: https://krishi.icar.gov.in/jspui/handle/123456789/31947.

## Author Contributions

AS conceptualized the idea. HB, PB, SG, MN, and RE conducted the field experiments. HB, AY, and CN carried out phenotyping of the germplasm accessions. HB and KV carried out the data analysis. HB and KV prepared the manuscript. HB, AS, KV, and SG edited the manuscript.

## Conflict of Interest

The authors declare that the research was conducted in the absence of any commercial or financial relationships that could be construed as a potential conflict of interest.

## Footnotes

1 http://archive.gramene.org/qtl/

2 http://qtaro.abr.affrc.go.jp/

3 https://rapdb.dna.affrc.go.jp/
